# *De novo* sequencing of *Bletilla striata* (Orchidaceae) transcriptome and identification of genes involved in polysaccharide biosynthesis

**DOI:** 10.1590/1678-4685-GMB-2019-0417

**Published:** 2020-06-26

**Authors:** Junfeng Niu, Guangming Zhao, Zeyuan Mi, Lijun Chen, Shuai Liu, Shiqiang Wang, Donghao Wang, Zhezhi Wang

**Affiliations:** 1 Shaanxi Normal University, College of Life Sciences, Key Laboratory of the Ministry of Education for Medicinal Resources and Natural Pharmaceutical Chemistry, National Engineering Laboratory for Resource Development of Endangered Crude Drugs in Northwest of China, Xi'an, Shaanxi, China

**Keywords:** Bletilla striata, transcriptome, germplasm resources, polysaccharide biosynthesis, qRT-PCR

## Abstract

*Bletilla striata* polysaccharide (BSP) is the main component of *Bletilla striata*, which has important pharmacological and pharmacological effects; however, due to the lack of genetic data, the metabolic pathways of BSP remain unclear. For this study, 11 representative resources of *B. striata* were analyzed, and the BSP contents of the different samples were significantly different; however, the monosaccharide composition of BSP was glucose and mannose. The representative samples were selected to observe their life history *in situ*, which were then divided and cultured in a greenhouse. Finally, samples from various organs of different plants were combined for transcriptome sequencing using the Illumina system. Our results summarized the BSP metabolic pathway, and we found that there were eight enzyme genes involved in biosynthesis, but these genes showed tissue specificity. Following qRT-PCR validation and comparative analysis, *manA* showed the highest expression; however, there were significant differences between the two germplasm resources in which the BSP content was significantly different, while *UGP2, GPI, PMM*, and *GMPP* had significant differences between the two samples. In summary, this study lays the foundation for further research into BSP metabolism and other physiological processes at the molecular level.

## Introduction

*Bletilla striata*, typically known as an important species of the *Bletilla* genus, is a significant medicinal compound for the treatment of many diseases, as recorded by Chinese pharmacopoeia (Chen *et al.*, 2018; Zhang *et al.*, 2019a). It is an ornamental flower but belongs to the Orchidaceae family (He *et al.*, 2017). Wild *B. striata* sources are primarily distributed across Yunnan, Shaanxi, Gansu, Hubei, and Zhejiang Provinces of China at elevations from 110-3,200 m (He *et al.*, 2017), as well as the Korean Peninsula and Japan (Chung *et al.*, 2013). At present, various constituents have been extracted and isolated from dried *B. striata* pseudobulbs, including polysaccharides, benzyl, phenanthrax, astragalus, terpenes, ethers, and anthocyanins (Guo *et al.*, 2016; Li *et al.*, 2018).

However, polysaccharides are one of the main components, which plays a variety of biological functions (Wang and Meng, 2015; Zhang *et al.*, 2019b). Research has revealed that BSP not only facilitates coagulation by inducing the expression of vascular endothelial growth factor (VEGF), but also prevent gastric ulcers by forming a protective film that coats the stomach wall, and is a common immune modulator. (Luo *et al.*, 2018, 2019; Zhang *et al.*, 2019a). The structure and function of BSP is closely related to its biological activity, which is often modified or cross-linked with other substances for use as a biomaterial in wound healing and drug delivery applications (Wang *et al.*, 2018). Although *B. striata* has numerous functions, data on its genetic makeup remains scar3ce, and the polysaccharide metabolism pathway is not clear. Therefore, it is necessary to conduct in-depth genetic research to discover its genes and their functionalitiess.

With the development and implementation of advanced DNA sequencing technologies, the biological metabolisms of active components may be discerned in medicinal plants (Fu *et al.*, 2019; Liang *et al.*, 2019; Niazian, 2019). There are few studies on the biosynthesis of polysaccharides (this research has focused primarily on fungal species) due to their unique structures and large molecular weights (Ribeiro *et al.*, 2012; Zhang *et al.*, 2019b). The biosynthesis pathways of *Ganoderma lucidum* polysaccharides provide a preliminary study of the various synthesis pathways of medicinal plants (Gao *et al.*, 2015).

The pharmacopoeia of the People's Republic of China has entered the genus *Polygonatum cyrtonema* and *Polygonatum sibiricum*, both of which employ polysaccharides as their key components, and their polysaccharide metabolic pathways have been elucidated (Wang *et al.*, 2017; Wang *et al.*, 2019a). It has been verified, via hot water extraction and ion exchange chromatography purification, that BSP is composed of glucose and mannose; however, the genetic data related to its polysaccharide biosynthesis is still unknown (Kong *et al.*, 2015).

For this study, comparative transcriptomes were analyzed using the roots, stems, leaves, flowers and seeds of *B. striata*. After determining the candidate genes involved in BSP biosynthesis, we selected the highest and lowest BSP germplasm contents, respectively, and verified the quality of the database and related genes involved in the BSP metabolic pathway by quantitative real-time PCR (qRT-PCR). The results of this study provided a theoretical basis for the study of *B. striata*.

## Materials and Methods

### Ethics statement

All experimental materials for this study were collected in China, but did not cause the species to be threatened or endangered. This research was conducted at the National Engineering Laboratory for Resource Development of Endangered Chinese Crude Drugs in Northwest China, Xi'an, China.

### Plant materials

The transcriptome-sequenced plants were collected from Zhen'an County, Shaanxi Province, in China on July 20, 2013. Once the plants were transplanted with soil at the greenhouse of National Engineering Laboratory for the Development of Endangered Medicinal Materials Resources in Northwest China, we separated apart the pseudobulbs of *B. striata* one by one for individual propagation, and finally the sample was divided into 11 plants. We chose to extract samples from the flowering to fruiting stage. At this time, the upper portion of the peduncle has a flower bud, whereas the lower part has a capsule that is formed by fertilization. Six samples were randomly selected for sampling. The roots, stems, leaves, flowers and fruits of different organs from all of the specific plants were collected equally and mixed together. After the samples (each about 2.0 g) were collected, they were flash-frozen with liquid nitrogen and stored at −80 °C.

Eleven regionally representative populations of *B. striata* were collected from 2014 to 2015, all of which were identified by Prof. Yaping Xiao (College of Life Science, Shaanxi Normal University). The origins of the rhizome samples collected from different germplasms are specified in Table S1, and all materials were grown in the laboratory germplasm field.

### Isolation, purification and detection of polysaccharides

The method of extracting the polysaccharides from *B. striata* rhizomes drew from the relevant articles previously published by our research group (Niu *et al.*, 2019). Once the fresh plant material was dried at 60 °C, it was ground to a powder and passed through a 60 mesh screen. The removal of lipids from the dry powder sample (100g) was achieved mixing it with petroleum ether, followed by twice extracting with distilled water (80 °C) for three hours (1:30, w: v). Once the extract was concentrated on a rotary evaporator, 95% alcohol (about a four-fold volume) was added and left overnight at 4 °C, after which the polysaccharide was precipitated and filtered. Following the removal of proteins using a repeated freeze-thaw method, the crude BSP polysaccharides were obtained via dialysis and lyophilization.

### BSP monosaccharide composition

GC-MS was employed to determine the composition of the BSP monosaccharides. The BSP sample (2.0 mg) was initially added to 2 M trifluoroacetic acid (TFA), and following hydrolyzation at 100 °C for 90 min, it was converted to alditol acetates. Subsequently, the acetylated structure was evaluated using an Agilent GC-MS QQQ7000 with a HP-5 fused silica capillary flame ionization detector.

The temperature gradient of the column was increased from a starting temperature of 160 °C at a rate of 4 °C min^−1^ to 250 °C, which was held for five min. The temperature of the injector and detector of the instrument was set to 250 °C, N_2_ was selected as the carrier gas and the flow rate was 1 mL min^−1^. The H_2_ flow rate and air flow rate were 30 mL min^−1^, and 400 mL min^−1^, respectively. The following standard monosaccharides were used as references: rhamnose, fucose, arabinose, xylose, mannose, galactose, and glucose.

### Establishment of library and sequencing (mRNA-Seq)

Transcriptome sequencing plants were collected from Zhen'an County, in Shaanxi Province. Mixed samples of collected roots, stems, leaves, flowers, and fruits were separately sequenced. The total RNA for each sample was extracted following the instructions of the total RNA Isolation Kit (TIANGEN, Beijing, China). High quality RNA was evaluated for library building using the HiSeq 2500 platform.

### *De novo* assembly and functional annotation

The Unigene library for this species was obtained by filtering rRNA and low-quality reads (two-terminal sequences) by data evaluation. Trinity software was used for the mixed assembly of sample data (Grabherr *et al.*, 2011). The most important transcripts were selected as unigenes (Mortazavi *et al.*, 2008), and Blastx (Altschul *et al.*, 1997) comparison was carried out with four common protein databases: NR (non-redundant protein database) (Pruitt *et al.*, 2005), Swiss-Prot Database (Apweiler *et al.*, 2004), KEGG Database (Kyoto Encyclopedia of Genes and Genomes protein Database) (Kanehisa *et al.*, 2004), and COGs (Clusters of Orthologous Groups) (Tatusov *et al.*, 2000). The functional annotation of the relevant unigene coding proteins was performed by comparing the data from proteins with similar results in the database.

### Analysis of differential gene expression

Firstly, we compared the sequencing results with the published Unigenes databas, using Bowtie software (version 1.1.2, JHU, Baltimore, MD, USA) (Langmead *et al.*, 2009). Secondly, RSEM (Li and Dewey, 2011) was performed to reflect the expression levels of different genes, and the abundance of corresponding transcripts was determined by calculating the values of RPKM (Reads Per Kilobase of transcript per Million mapped reads) (Trapnell *et al.*, 2010). Finally, DESeq software (Anders and Huber, 2010) was used to further compare the gene expression patterns from different germplasm resources, SXZA and AHXC, and five different organs: roots, stems, leaves, flowers and fruits.

### Identification of genes related to BSP biosynthetic pathway

Through the annotation of unigenes in the KEGG database, the genes involved in the polysaccharide metabolism pathway were summarized as candidate genes. Candidate genes for further reference included KEGG metabolic pathways of starch and sucrose metabolism, as well as amino sugar and nucleotide sugar metabolism on the metabolic pathways involved in the gene's comments. As a result, further screening participated in the reaction of related enzymes, where all of the enzymes and *Arabidopsis thaliana* annotated information in the database, combined with Swiss-Prot, and a further annotated database relating to enzyme glycosyl transferase (GTs, Glycosyltransferases).

### Real-time PCR

We selected *GAPDH* as the reference gene, and a total of eight candidate genes were screened, where the related primers being designed using Premier 3.0, with all of the above presented in Table S2. The total RNA was extracted from two germplasm groups, SXZA and AHXC, as well as five different organs, roots, stems, leaves, flowers, and fruits. Reverse transcription to cDNA was performed according to the instructions of Takara's Prime-Script^TM^ RT Master Mix (TaKaRa, Kyoto, Japan).

Real-time PCR experiments were performed on a Light Cycler 96 Instrument (Roche, Germany) according to the 2×Sybr Green qPCR Mix instructions (Aidlab, Beijing, China). The relative expression was calculated using the 2^−ΔΔCt^ method (Schmittgen and Livak, 2008). All qRT-PCR experiments involved in this study underwent three biological and three technical replications.

### Statistical analysis

All experimental results in this study were expressed as a mean standard deviation (SD), using one-way analysis of variance (ANOVA) of DPS, and Duncans multivariate test was performed. Values were considered statistically significant, when p < 0.05.

## Results

### BSP composition and *B. striata* germplasm (SXZA) content

**Figure 1:**
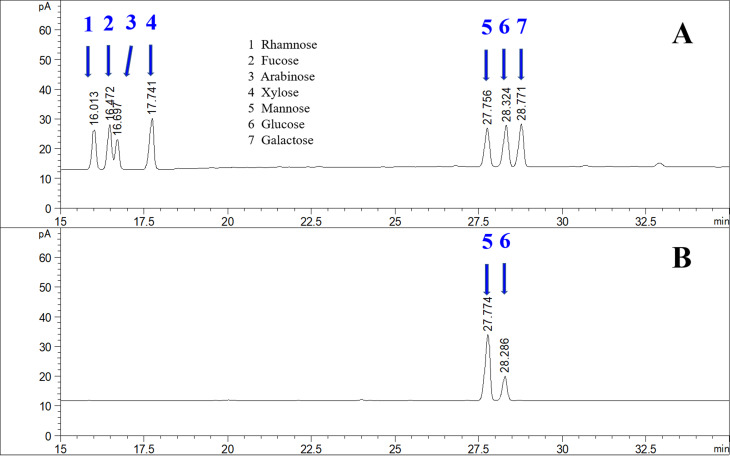
BSP composition of *B. striata* germplasm SXZA. (A) Monosaccharide standards, arrows 1-7 represent rhamnose, fucose, arabinose, xylose, mannose, glucose, and galactose, respectively. (B) Arrows 5 and 6 represent mannose and glucose.

Water-soluble polysaccharides were extracted from the dried rhizomes of the *B. striata* germplasm (SXZA), which were collected from Zhen'an County, in Shaanxi Province, China. As shown in Figure 1, the content of BSP was 22.85 ± 0.04%, which was comprised of glucose and mannose, and the mole ratio was 0.745:0.255 by gas chromatography. We observed the life history of the whole plant from 2012 to 2013 on the original land (E109°9’11″, N33°25’33″). The plants were segmented into multiple plants by pseudobulbs, and the flowering to fruiting stage was further selected. Transcriptome sequencing was performed by mixing the different organs of different plants.

### Polysaccharide yields from different germplasm resources

A total of 11 germplasm resources of *B. striata* were collected from Shaanxi, Anhui, Hubei, Sichuan, and Henan Provinces from 2013 to 2015, and the polysaccharide content were analyzed between the different samples. As shown in Table 1, the highest polysaccharide content was 36.48 ± 0.27% in Shangzhou, of Shaanxi Province, whereas the lowest sample was 20.56 ± 0.04% in Xuancheng, of Anhui Province.

**Table 1:** The polysaccharide content of *B. striata* in different germplasm resources.

No.	Region	polysaccharide yield
1	Shangzhou, Shaanxi province (SXSZ)	36.48 ± 0.27 aA
2	Liuba, Shaanxi province (SXLB)	27.2 ± 0.35 deD
3	Lvyang, Shaanxi province (SXLY)	31.450.25 bB
4	Ningqiang, Shaanxi province (SXNQ)	26.580.16 deD
5	Qichun, Hubei province (HBQC)	29.79 ± 0.04 cC
6	Baokang, Hubei province (HBBK)	27.18 ± 0.38 dD
7	Mianxian, Shaanxi province (SXMX)	26.5 ± 0.03 eD
8	Zhenan, Shaanxi province (SXZA)	22.85 ± 0.04 gF
9	Lushi, Henan province (HNLS)	25.56 ± 0.03 fE
10	Xuancheng, Anhui province (AHXC)	20.56 ± 0.04 hG
11	Bashan, Anhui province (AHBS)	22.77 ± 0.05 gF

BSP content is significantly different between germplasms at p < 0.05 (different lowercase letters) and p < 0.01 (different capital letters).

### Transcriptome sequencing, *de novo* assembly, and assessment of assembly program

Using the Illumina sequencing system, we obtained 11.4 Gb of clean data from five mixed samples. The clean data and Q30 values of each sample were greater than 2.04 Gb and 93.26% (Table 2). Following the removal of the adapter sequence, ambiguous reads, and low-quality reads in order, the remaining data was compiled using Trinity software, where after 4,784,551 contigs were obtained and the N50 was 48 bp. With clustering contigs based on pairing information and the similarity of overlapping sequences, we obtained 115,004 transcripts and the N50 was 1,927bp. The most important transcripts were selected as unigenes, where not only were 58,397 unigenes obtained, but their N50 attained 1,385 bp. The lengths greater than 1,000 bp and 500 bp accounted for 23.97% and 42.54%, respectively (Table 3).

**Table 2:** Output statistics of *B. striata* transcriptome sequencing.

Samples	Tissues	Raw Reads	Raw Dates (G)	GC percentage	N percentage	Q30 percentage
*B. striata*	Leaves	7,740,447	2.07	46.52%	0.00%	93.26%
	Stems	7,909,965	2.04	46.20%	0.00%	93.35%
	Roots	8,289,337	2.15	46.32%	0.00%	93.44%
	Flowers	9,896,931	2.54	47.19%	0.00%	93.31%
	Seeds	10,280,142	2.60	46.12%	0.00%	93.49%

The roots, stems, leaves, flowers and seeds of the sequencing samples were collected from the same germplasm SXZA.

**Table 3:** Output statistics from assembly of *B. striata* transcriptome sequencing.

Length range (bp)	Number of contigs	Transcript abundance	Number of unigenes
0-300	4,738,699 (99.04%)	23,017 (20.02%)	20,023 (34.30%)
300-500	17,220 (0.36%)	17,967 (15.62%)	13,520 (23.16%)
500-1,000	13,849 (0.29%)	21,495 (18.69%)	10,842 (18.57%)
1,000-2,000	10,069 (0.21%)	30,052 (26.13%)	8,896 (15.24%)
> 2,000	4,714 (0.10%)	22,473 (19.54%)	5,098 (8.73%)
Total number	4,784,551	115,004	58,376
Total length	242,503,081	138,028,967	45,367,096
N50 length	48	1,927	1,385
Mean length	50.68	1200.21	777.11

### Functional annotations

All unigenes generated by the Illumina platform were compared with NR, Swiss-Prot, GO, Subsequently, amino acid sequences were predicted and, unigenes were compared with the Pfam database to obtain annotation information. In these public databases, a total of 33,344 unigenes (57.10%) were consistent with the homologous sequences. Among them, 13,232 (22.66%) were 300 to 1,000 bp long while 13,224 (22.64%) were longer than 1,000 bp (Table 4).

**Table 4:** Summary statistics of functional annotations for *B. striata* unigenes via public databases.

Database	Annotated number	300<=length< 1,000	Number longer than 1,000 bp
COG	11,282 (19.32%)	3,664 (6.27%)	5,720 (9.805)
GO	21,036 (36.02%)	7,730 (13.24%)	9,406 (16.11%)
KEGG	7,736 (13.25%)	2,910 (4.98%)	3,314 (5.67%)
Swissprot	21,050 (36.05%)	7,857 (13.45%)	9,855 (16.88%)
NR	33,239 (56.92%)	13,189 (22.59%)	13,221 (22.64%)
All_Annotated	33,344 (57.10%)	13,232 (22.66%)	13,224 (22.64%)

When compared with the NR database, approximately 56.92% (33,239) of the unigenes were successfully annotated. Among these unigenes, 17.80% had the best matches to the *Piriformospora indica* sequences.

**Figure 2:**
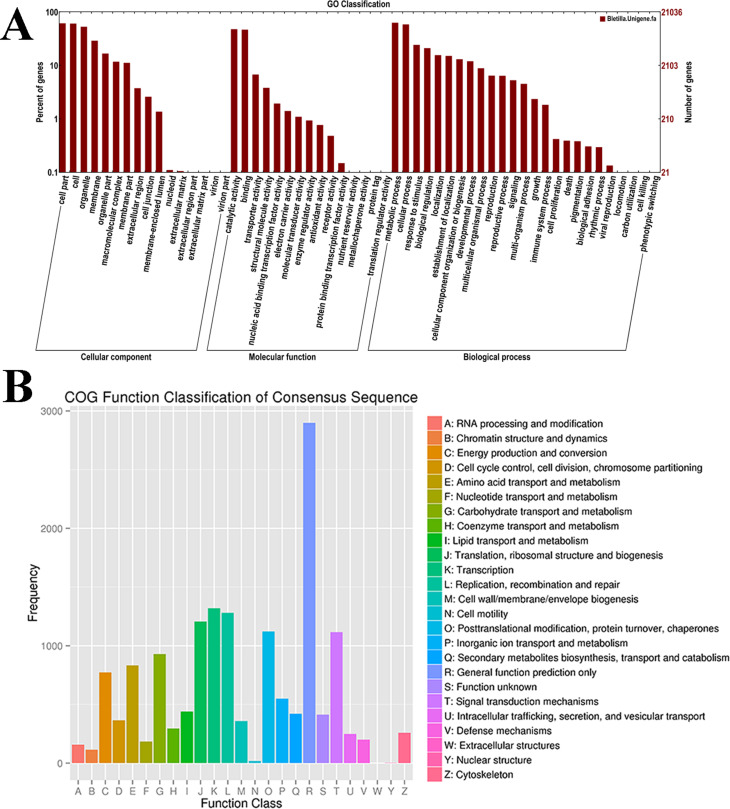
GO and COG functional annotations of *B. striata* transcriptome. (A) GO classification results for annotated unigenes in *B. striata*. The *B. striata* unigenes were annotated using BLAST searches against the public databases GO, and the results were categorized and viewed using WEGO. Percentage of genes (y-axis) indicates the proportion of *B. striata* unigenes that have relevant GO annotations in the three major GO domains “cellular components”, “molecular function”, and “biological process”. (B) The *B. striata* unigenes were compared with the public database COG using BLAST software to obtain annotation information, the abscissa represents the contents of each classification, and the ordinate represents the number of genes.

Unigenes that completed the NR database comparison were further compared with the GO database, and 21,036 (36.02%) unigenes were annotated and assigned to 56 functional groups based on the GO terms annotations (Figure 2A). Among these unigenes related to “cell components”, 12,799 (60.84%) were classified as “cell” (GO: 0005623), followed by “organelles” (GO: 0043226; 11,166 or 53.08%). However, among the unigenes related to “molecular function”, 47.69% were classified as catalytic activity (GO: 0003824; 10,032) and 46.73% were classified as binding activity (GO: 0005488; 9,830). The GO terms of biological processes were divided into “cellular process” (GO: 0009987; 58.58% or 12,322) and “metabolic process” (GO: 0008152; 63% or 13,254).

By comparing the transcriptome data of *B. striata* with the public COG database, the function and classification of unigenes were predicted. In general, 11,282 unigenes (19.33%) were divided into 24 categories (Figure 2B). Among these, the largest functional group was “General function prediction only” (2,898, 25.29% of the COG annotations), followed by “Replication, recombination and repair” (1,280, 11.35%) and “Translation, ribosomal structure and biogenesis” (1,206, 10.69%).

Finally, through comparisons with four public protein databases, we obtained 58,063 CDSs, which accounted for 99.46% of all identified unigenes. The length distribution of CDSs revealed that 21,443 were from 200 to 1,000 bp long, and 25.82% were longer than 500 bp. In addition, 5,847 (10.07%) were from 1,000 to 2,000 bp long, 1,353 (2.33%) were 2,000 to 3,000 bp long, and 557 (0.96%) were longer than 3,000 bp (Figure S1).

### Identification of transcription factors

When compared with the plant transcription factor database (Jin *et al.*, 2014), we obtained 13,764 unigenes that had a good matching coefficients (identity >80%). These unigenes belonged to 59 families of plant transcription factors (Table 5), of which the most abundant unigenes were bHLH, NAC, MYB-related, MYB, WRKY, FAR1, B3, ERF, C2H2, and bZIP families.

**Table 5:** Putative transcription factors in *B. striata* unigenes.

TF family	NU	NTP	Percentage (%)	TF family	NU	NTP	Percentage (%)
bHLH	1350	11428	0.12	Nin-like	204	1002	0.20
MYB	809	8746	0.09	HB-other	215	987	0.22
ERF	589	8688	0.07	NF-YA	98	943	0.10
NAC	889	8133	0.11	ARR-B	33	914	0.04
C2H2	589	7336	0.08	WOX	117	903	0.13
MYB_related	844	6410	0.13	CO-like	59	854	0.07
bZIP	526	6258	0.08	DBB	35	764	0.05
WRKY	654	5936	0.11	GRF	34	752	0.05
B3	598	4051	0.15	YABBY	176	725	0.24
C3H	480	4019	0.12	E2F/DP	168	692	0.24
G2-like	452	3935	0.11	GeBP	120	683	0.18
GRAS	393	3915	0.10	BES1	61	651	0.09
HD-ZIP	373	3436	0.11	CPP	77	594	0.13
M-type	254	2978	0.09	EIL	33	531	0.06
MIKC	217	2864	0.08	CAMTA	80	518	0.15
LBD	143	2779	0.05	SRS	34	506	0.07
Trihelix	257	2599	0.10	BBR-BPC	40	492	0.08
FAR1	633	2542	0.25	LSD	22	402	0.05
Dof	109	2312	0.05	RAV	36	289	0.12
GATA	186	2229	0.08	Whirly	12	233	0.05
ARF	261	1914	0.14	VOZ	32	227	0.14
HSF	170	1833	0.09	NF-X1	46	176	0.26
TALE	276	1797	0.15	HB-PHD	33	160	0.21
AP2	89	1776	0.05	S1Fa-like	161	158	1.02
TCP	218	1704	0.13	LFY	5	100	0.05
SBP	88	1675	0.05	HRT-like	13	95	0.14
NF-YB	172	1334	0.13	STAT	56	84	0.67
ZF-HD	44	1066	0.04	SAP	2	63	0.03
NF-YC	97	1018	0.10	NZZ/SPL	2	45	0.04

NU, number of unigenes; NTP, number of TF genes in PlantTFDB database; percentage = NU//NTP.

### KEGG pathway analysis

To further elucidate the biological metabolic processes of *B. striata*, we obtained 7,736 unigenes which were involved 112 metabolic pathways based on a comparison with the KEGG database (Table S3).

Among these unigenes, 529 were related to “ribosome pathways”, 364 played the “Oxidative phosphorylation pathways” function, 276 were involved in “Protein processing in endoplasmic reticulum”, 254 for “Protein processing in endoplasmic reticulum Spliceosome”, and 253 for “Glycolysis/Gluconeogenesis” (Table 6). Through analysis, a total of 1,364 unigenes were mapped to related metabolic pathways of carbohydrates, which mainly involved metabolic pathways such as glycolysis/gluconeogenesis, starch and sucrose metabolism, pyruvate metabolism, and amino sugar and nucleotide sugar metabolism (Table 7).

**Table 6:** The 20 most-represented KEGG pathways (KO, KEGG Orthology).

Metabolism pathway	Number of unigenes	(KO) entry
Ribosome	529 (6.17%)	ko03010
Oxidative phosphorylation	364 (4.25%)	ko00190
Protein processing in endoplasmic reticulum	276 (3.22%)	ko04141
Spliceosome	254 (2.96%)	ko03040
Glycolysis / Gluconeogenesis	253 (2.95%)	ko00010
Purine metabolism	222 (2.59%)	ko00230
RNA transport	222 (2.59%)	ko03013
Starch and sucrose metabolism	207 (2.42%)	ko00500
Plant hormone signal transduction	179 (2.09%)	ko04075
Carbon fixation in photosynthetic organisms	172 (2.01%)	ko00710
Ubiquitin mediated proteolysis	163 (1.90%)	ko04120
Pyrimidine metabolism	158 (1.84%)	ko00240
Pyruvate metabolism	158 (1.84%)	ko00620
Amino sugar and nucleotide sugar metabolism	153 (1.79%)	ko00520
Phagosome	149 (1.73%)	ko04145
Arginine and proline metabolism	147 (1.72%)	ko00330
Citrate cycle (TCA cycle)	144 (1.68%)	ko00020
Plant-pathogen interaction	144 (1.68%)	ko04626
Peroxisome	142 (1.61%)	ko04146
Endocytosis	138 (1.56%)	ko04144

**Table 7:** The number of unigenes involved in polysaccharide biosynthesis in *B. striata* database.

Metabolism pathway	Number of unigenes	(KO) entry
Glycolysis / Gluconeogenesis	253	ko00010
Starch and sucrose metabolism	207	ko00500
Pyruvate metabolism	158	ko00620
Amino sugar and nucleotide sugar metabolism	153	ko00520
Citrate cycle (TCA cycle)	144	ko00020
Pentose phosphate pathway	105	ko00030
Fructose and mannose metabolism	97	ko00051
Pentose and glucuronate interconversions	81	ko00040
Galactose metabolism	64	ko00052
N-Glycan biosynthesis	58	ko00510
Glycosylphosphatidylinositol(GPI)-anchor biosynthesis	18	ko00563
Other glycan degradation	15	ko00511
Glycosaminoglycan degradation	11	ko00531

### Candidate genes involved in BSP biosynthesis

To better understand the metabolic pathways of BSP, referring to the annotation information on the KO 00500 and KO 00520 metabolic pathways in the KEGG database, we determined the key enzyme genes involved in these pathways (Table 8).

**Table 8:** Unigenes related to the biosynthesis of polysaccharide in *B. striata.*

Enzyme code	Enzyme name	Abbreviation	Number
2.7.1.1	hexokinase	*HK*	8
2.7.1.4	fructokinase	*scrK*	12
5.3.1.9	glucose-6-phosphate isomerase	*GPI*	6
5.4.2.2	phosphoglucomutase	*pgm;*	13
2.7.7.9	uridine-diphosphate glucose pyrophosphorylase	*UGP2*	1
5.3.1.8	mannose-6-phosphate isomerase	*manA*	3
5.4.2.8	phosphomannomutase	*PMM*	4
2.7.7.13	mannose-1-phosphate guanylyltransferase	*GMPP*	6

The precursors of all plant polysaccharides were nucleoside-bisphosphate-sugar (NDP-sugar). Under the action of different glycosyltransferases (GTs), NDP-sugars were catalyzed to increased sugar chains to form new polysaccharides (Gutmann *et al.*, 2017; Yuan *et al.*, 2018a).

The results indicated that the polysaccharides of *B. striata* were derived from glucose and mannose; therefore, the metabolic processes of UDP-glucose (UDP-Glc) and GDP-mannose (GDP-Man) were the key metabolic pathway of BSP. UDP-Glc and GDP-Man were derived from glucose-6-phosphate (Glc-6P) and fructose-6-phosphate (Fru-6P), respectively. Under the action of NDP-sugar interconversion enzymes (NDP-sugar conversion enzymes), they carried out the next reaction.

Referring to previous research progress on polysaccharide metabolism (Liepman *et al.*, 2010; Wang *et al.*, 2019a; Zhang *et al.*, 2019b), the plant polysaccharide pathway consisted of three steps. Firstly, sucrose was converted to Glc-6P and fructose under the action of β-fructofuranosidase (encoded by *sacA*) and phosphotransferase system (encoded by *scrA*). Fructokinase (encoded by *scrK*) and hexokinase (encoded by *HK*) were also involved in the biosynthesis of fructose to Fru-6P. Under the action of related enzymes, Glc-6P and Fru-6P were formed into UDP-Glc and GDP-Man respectively (Zhang *et al.*, 2017; Geiger *et al.*, 2019), which were glucose phosphotransase (encoded by *pgm*), uridine diphosphate glucose pyrophosphorylase (*UGP2*), glucose-6-phosphoisomerase (*GPI*), mannose-6-phosphoisomerase (*manA*), mannose phosphate enzyme (*PMM*), and mannose-1-phosphotransferase (*GMPP*).

**Figure 3:**
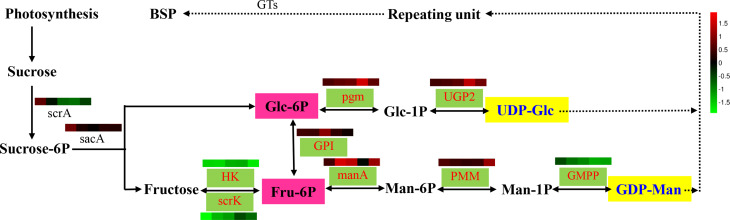
Proposed pathways for polysaccharide biosynthesis in *B. striata*. Activated monosaccharide units, marked in blue with yellow background, key enzymes, marked in red with green background; and bolded text with pink background indicates key intermediates. From left to right, the colors represent leaves, stems, roots, flowers, and seeds organization, respectively. The full line arrows represent the identified enzymatic reactions, and the dashed line arrows represent multiple enzymatic reactions by multiple steps.

Secondly, under the action of a series of enzymes, UDP-Glc formed NDP-sugars. The main pathways were UDP-Glc, which formed NDP-sugars through a series of enzymes. Thirdly, NDP-sugars were added to various sugar residue chains to form polysaccharide complex units under the action of glycosyltransferases (Figure 3).

In the first step reaction, glucose and fructose were converted under the action of a series of kinases, and the related enzymes included hexokinases (*HKs*) and fructokinases (*scrKs*). By comparison, we concluded that 537-bp Unigene 33226 was a *HK* homologue with the highest amino acid homology (93%) to *HK* in *Dendrobium catenatum* (Sequence ID: XM_020834402.2) (Figures S2, S3). In addition, the 1,488-bp Unigene 44091 was consistent in homology with the *scrK* from *D. catenatum* (Sequence ID: XM_020847040.2) (Figures S2, S4).

The results of the comparative transcriptome in Table 8 were found in the stem of the main accumulation site of BSP, and directed from Glc-6P through the intermediate product Fru-6P, Man-6P, and Man-1P. Eventually, GDP-Man, and related enzymes *GPI, manA, PMM*, and *GMPP* exhibited extremely strong activity, which means that the biotransformation process, from Glc-6P to GDP-Man may have been its primary metabolic pathway.

Unigenes involved in BSP metabolism were expressed by the RPKM value in transcriptome database. Under general conditions, the unigene with the maximum RPKM value was selected as the gene expression quantity (Table 9).

**Table 9:** Unigenes expression levels in BSP biosynthesis calculated by the method of RPKM.

Gene	Enzyme name	Roots	Stems	Leaves	Flowers
*GAPDH*	glyceraldehyde-3-phosphate dehydrogenase	46.33	52.52	161.32	25.62
*GMPP*	mannose-1-phosphate guanylyltransferase	12.49	21.32	18.87	10.44
*PMM*	phosphomannomutase	45.4	105.53	65.89	70.58
*manA*	mannose-6-phosphate isomerase	118.54	446.52	57.38	41.65
*HK*	hexokinase	9.4	8.36	6.3	10.42
*scrk*	fructokinase	10.81	11.52	4.44	24.06
*GPI*	glucose-6-phosphate isomerase	82.38	106.15	57.53	66.08
*pgm*	phosphoglucomutase	67.89	144.18	63.33	248.17
*UGP2*	uridine-diphosphate glucose pyrophosphorylase	59.08	123.13	56.87	208.29

Depending on the abundance of the transcribed RNA-seq assay, we selected eight candidate unigenes; *GMPP, PMM, manA, HK, scrK, GPI, pgm* and *UGP2.* In the database, the least consistent with their homologs was *scrK* (Unigene 44,091; 98% identity), followed by *PMM* (Unigene 36,348; 99%), *manA* (Unigene 45,816; 99%), and *GPI* (Unigene 32,627; 99%). The remaining identity of the unigenes was 100%, which were *GMPP* (Unigene 39,307), *HK* (Unigene 33,226), *pgm* (Unigene 46,080) and *UGP2* (Unigene 31559). All these details were aligned as shown in Table S4, which confirmed that the selection of unigenes related to BSP metabolism was quite conservative.

It can be seen from Table 8 that the expression levels of related enzyme genes in the roots, stems, leaves, and flowers of *B. striata* were different, among which *GMPP, manA* and *GPI* had the highest expression levels in stems. Both *pgm* and *UGP2* were the most expressed in flowers, while *PMM, HK*, and *scrK* were most expressed in seeds.

### Analysis of BSP biosynthetic pathway

In combination with the database annotation of the polysaccharide metabolic pathway and the experimental results of the monosaccharide composition of *B. striata*, we obtained the potential biosynthesis pathways for BSP formation from sucrose.

The BSP synthesis pathway primarily consisted of three steps. Firstly, under the action of related enzymes such as *UGP2, pgm, GPI, manA, PMM*, and *GMPP*, Glc-6-p and Fru-6-p formed UDP-Glc and GDP-Man, respectively. Secondly, the NDP-sugars were added to sugar residues to form complex polysaccharide units. Therefore, in the BSP metabolic pathway, UDP-Glc and GDP-Man were formed mainly by the key enzyme genes in step one, and finally complex units were formed through the NDP-sugars by NSEs in step two. Thirdly, under the action of related GTs, the NDP-sugars were added to the polysaccharide. The logarithms of the RPKM values of different tissues in the metabolic pathway were expressed as different colors (from left to right, the colors represent leaves, stems, roots, flowers, and seeds). Here, a normalized data processing method was used and the drawing was completed on a heat map generated by the R software (Figure 3).

### Quantitative real-time PCR analysis

**Figure 4:**
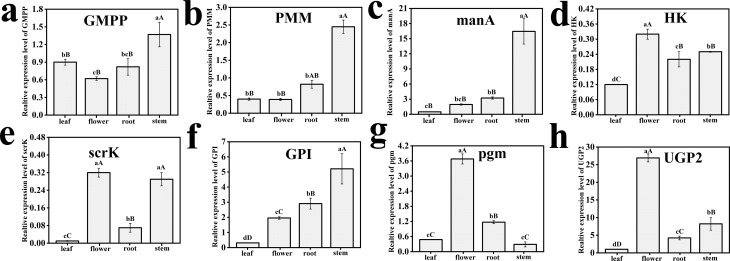
Expression patterns of some novel transcripts related to polysaccharide biosynthesis in *B. striata*. (a-h) expression patterns of key enzyme genes *GMPP, PMM, manA, HK, scrK, GPI, pgm*, and *UGP2* in the BSP pathway.

Quantitative real-time PCR can reflect the expression abundance of related genes in the BSP metabolic pathway, and we selected *GAPDH* as the experimental reference. We screened the expression patterns of *GMPP, PMM, manA, HK, scrK, GPI, pgm*, and *UGP2* genes in the BSP metabolic pathways for different organs of *B. striata* (SXZA). According to the expression values of related genes in various tissues, *HK, pgm*, and *UGP2* had the largest content in flowers, while *GMPP, manA*, and *GPI* had the highest content in stems. These genes may play an important role in the biosynthesis of BSP (Figure 4).

**Figure 5:**
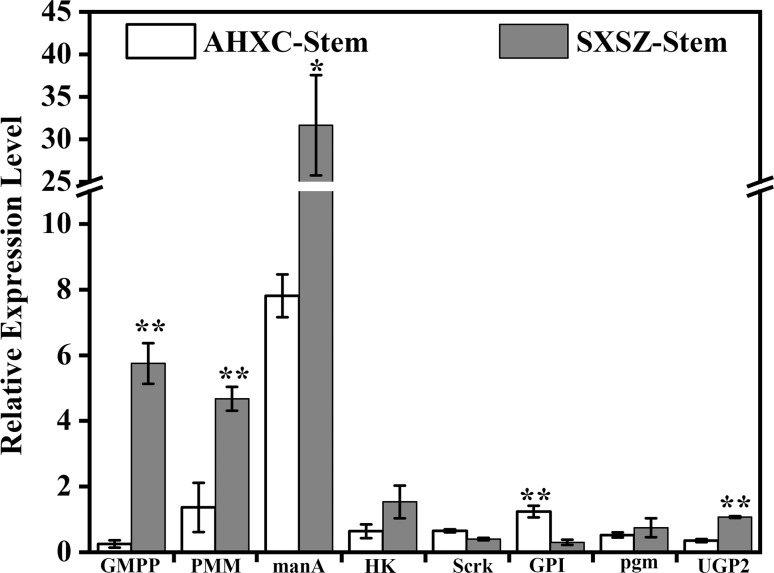
The expression of candidate unigenes involved in BSP biosynthesis between different germplasm resources. AHCX and SXSZ represent samples collected from Xuancheng No. 218 in Anhui Province and Shangzhou No. 82 in Shaanxi Province, respectively.

We further compared the expression patterns of different transcripts with the RPKM values of related genes in the transcriptome sequencing data group. *GMPP, scrK, GPI,* and *UGP2* had the same expression patterns in the two groups of data, while the expression of the remaining four genes was inconsistent. Futher, according to the polysaccharide yield analysis of different germplasm resources, we selected the fresh plant pseudobulb (SXSZ-82 and AHXC-218) germplasm resources for experiments to verify the expression levels of related genes in different tissues and parts. Compared with the reference gene, it was concluded that there were no significant differences in expression level quantities between *HK, scrK*, and *pgm*. It was observed that *manA* had the highest expression between the two species; however, there were significant difference between them, while *UGP2, GPI, PMM*, and *GMPP* had considerable differences between the two samples (Figure 5).

## Discussion

To date, this is the first time that *De novo* sequencing of the different organs of *B. striata* using the Illumina HiSeq 2500 platform explored the metabolic pathways of polysaccharides. After completing the assembly, we obtained 11.4 Gb of clean data and 58,397 unigenes, which made it possible to study the BSP pathway at the molecular level. Compared to the published databases of other medicinal orchid plants, *B. striata* had more unigenes than did *Dactylorhiza hatagirea* (37,371) and *Gastrodia elata* (56,884), but fewer than *Dendrobium huoshanense* (478,361), *Cymbidium goeringii* (85,868), and *Dendrobium officinale* (145,791) (Yuan *et al.*, 2018b; Dhiman *et al.*, 2019; Wang *et al.*, 2019b).

The average unigene length was 777.11 bp, which was longer than *D. hatagirea* (551.10 bp) and *D. huoshanense* (764.73 bp), but less than *C. goeringii* (1,194 bp) and *G. elata* (844.34 bp). In our experimental results, 23.97% of the sequence length was greater than 1,000bp, which indicated that the use of the Illumina technology platform had a higher assembly quality and stability in the research of medicinal plants.

There have been published studies on the overall transcriptome of the *B. striata* plant over its entire developmental stage. The transcriptome sequencing of samples from the protocorm stage to root, stem, leaf, and pollenated capsule stages were performed, and 127,261 overlapping unigenes were identified with mean unigene lengths of 612 bp. The authors successfully screened five markers using EST-SSR technology for their identification with their relatives (Xu *et al.*, 2018).

With the advent of the big data era and the development of omics technology, increasing numbers of studies on polysaccharide metabolic pathways have been conducted. Through the transcriptome analysis of medicinal plants *Codonopsis pilosula* using high-throughput sequencing technologies, the biosynthetic pathways of codonopsis polysaccharides were summarized and verified, which initially elaborated the polysaccharide metabolic pathways of medicinal plants (Gao *et al.*, 2015).

Similarly, polysaccharide is the main medicinal component of the *Polygonatum* genus. The polysaccharide metabolism pathways of *P. cyrtonema* and *P. sibiricum* were thoroughly investigated, which were consistent and comprised of three steps. However, due to the differences in monosaccharide composition and content, the expression of some key enzyme genes in the polysaccharide metabolic pathway may be different (Wang *et al.*, 2017; Wang *et al.*, 2019a). The genome sequencing of *D. candidum* was completed, which provided ample support for the study of polysaccharide metabolism pathways in other orchids (Zhang *et al.*, 2016a; Zhang *et al.*, 2016b).

Through related bioinformatics software analysis, the candidate unigenes of the BSP metabolic pathway were identified. According to previous experimental results, the BSP composition was glucose and mannose; however, the molar ratio was different (Figure S5). Therefore, what was of more interest was the conversion between glucose and mannose, and the further formation of polysaccharide coincidence units. We further analyzed the expression levels of related genes in the polysaccharide pathway in different *B. striata* tissues and concluded that the expression levels of *manA* and *UGP2* were the highest (Figure 4).

As is well recognized, *UGP2* is the key enzyme gene for glucose-1-phosphate to form UDP-Glc, while *manA* is the key enzyme for fructose-6-phosphate to form mannose-6-phosphate in plants. The expression level of *manA* in plants was 3.36 times that of *UGP2*, while the molar ratio of mannose to glucose in the same material of *B. striata* was 2.91:1. The expression levels of the two key genes mentioned above were approximately consistent with the composition ratio of the two monosaccharides, and it is predicted that there may be some relationship between them, which requires verification through further experimentation.

The main component polysaccharide of *B. striata* accumulates in the pseudobulb (Liao *et al.*, 2019). We can observe from Figure 5 that *manA* has the highest expression in the stems of SXSZ-82 and AHXC-218 sample; however, the expression of the former is four times that of the latter. According to the experimental results in Table 1, the polysaccharide yield of the SXSZ-82 sample was 36.48%, which was much larger than 22.77% in the AHXC sample. While *manA* is a key gene inducing Glc-1P to the Man-6P pathway, we can understand that there should be a correlation between high-activity samples and highly expressed genes. The further focus would be the gene expression of key enzymatic polysaccharide pathways, and the study of their correlations between enzyme activity and the BSP content.

*GMPP* also showed strong biological activity in the *B. striata* polysaccharide metabolic pathway, as a key enzyme gene in the pathway, *GMPP* plays an important role in the accumulation of GDP-Man in plants. Similarly, in *A. rabidopsis thaliana*, the establishment of mutants and a yeast hybrid experiment also confirmed that *GMPP* was the rate-limiting enzyme synthesized by GDP-Man (Sawake *et al.*, 2015). The same conclusion was also drawn in the study of the polysaccharide metabolic pathway of *P. sibiricum* (Wang *et al.*, 2017). In the process of studying the polysaccharide metabolism pathway, a complex network structure is formed due to the variety of monosaccharide components. It is possible to further implement such work by selecting species that are similar in type to BSP, have a small variety of structures and a well-defined overall structure.

Although the transcriptomes of different organs of *B. striata* were sequenced in this study, its bioinformatics database was established, and the enzyme genes in relevant BSP metabolic pathways were summarized. However, the research on *B. striata* is primarily focused on pharmacology, and there are relatively few studies on its molecular biology. The complexity of the BSP structure makes it difficult to study its monosaccharide composition. Therefore, future research should focus on verifying the functional prediction of related genes through modern molecular biology and proteomics techniques.

## Conclusions

As a common Chinese medicinal plant, *B. striata* has a variety of pharmacological effects. In this study, a bioinformatics database was established by performing transcriptome analysis on different organs of *B. striata*. Our experimental results provided useful information for studying the BSP metabolic pathway of this species, and the functions of several genes were confirmed by qRT-PCR. These research results not only contribute to laying the foundation for further elaborating the biosynthesis of related polysaccharides, but also provide candidate genes for metabolite synthesis, which can be employed for the evaluation of germplasm resources in *B. striata*.

## Supplementary Material

The following online material is available for this article:

Figure S1 Distribution of lengths for CDSs predicted from *B. striata* unigenes.

Figure S2 Trees produced from Neighbor-joining phylogenetic analysis of homologs for *HK* and *scrK* in various plant species.

Figure S3 Alignment of *HK* between *B.letilla striata* and *D.endrobium catenatum*.

Figure S4 Alignment of *scrK* between *B.letilla striata* and *D.endrobium catenatum*.

Figure S5 GC-MS map of monosaccharide composition of BSP in different germplasm resources.

Table S1 Origins of rhizome samples collected from different germplasms of *B.letilla striata*.

Table S2
*B.letilla striata* genes and primers used in real-time PCR.

Table S3 Number of unigenes from *B. striata* assigned to KEGG reference pathways.

Table S4 Identities of candidate unigenes related to BSP biosynthesis, analyzed at nucleotide level.
