# Supplementary material for: De novo sequencing of Bletilla striata (Orchidaceae) transcriptome and identification of genes involved in polysaccharide biosynthesis
Source: Genet Mol Biol. 2020 Jun 26;43(3):e20190417. doi: 10.1590/1678-4685-GMB-2019-0417 (PMC7315133; doi:10.1590/1678-4685-GMB-2019-0417)
Supplement: Supplementary file 1 [file 1415-4757-GMB-43-3-e20190417-suppl6.pdf]

Supplementary Material to "*De novo* sequencing of *Bletilla striata* (Orchidaceae) transcriptome and identification of genes involved in polysaccharide biosynthesis"

Table S2 – *B. striata* genes and primers

Gene	Unigene	Primer sequences（5'-3'）
BsGAPDH	c42693.graph_c0	GCT AAA GAC GCT GTC ACT GAG
		GAG ACC AGA AAC TTA CCC TCG
BsGMPP	c39307.graph_c0	GGT GAA CCT TTC TTT GTG CTC
		TCT CTT CCA TTA CCA CGA CAC
BsPMM	c36348.graph_c0	TCA CGC TCC ATT ACA TTG CT
		TTC CTC TTG GCT ACA GTT GC
BsmanA	c45816.graph_c0	GGA GAG AAG GTT GTG AAG AGG
		CTT ATG AAG CAA TCC AGC CA
BsHK	c33226.graph_c1	AAG ATG TTC CTT TGA GGG CTC
		GCA ATC ACA GTT CTC CTG GA
Bsscrk	c44091.graph_c0	AAC CAC CGA GCA ACT GTT TG
		CCA CTT GAT GAG AAT GCC TG
BsGPI	c32627.graph_c0	TTT GGA CTA CTC TCG GCA GT
		CCA CAT TCC TCC CAT CAC TA
Bspgm	c46080.graph_c0	TTA CTG CGG AGA AAG TGA GAG
		CAC GGA TAA CAC AAG ATA CAG C
BsUGP2	c31559.graph_c0	CAT ACT TTC AAC CAG AGC CA
		GAA GAC ATA CTC CTT TCC CTG AG
