# Supplementary material for: De novo sequencing of Bletilla striata (Orchidaceae) transcriptome and identification of genes involved in polysaccharide biosynthesis
Source: Genet Mol Biol. 2020 Jun 26;43(3):e20190417. doi: 10.1590/1678-4685-GMB-2019-0417 (PMC7315133; doi:10.1590/1678-4685-GMB-2019-0417)
Supplement: Supplementary file 2 [file 1415-4757-GMB-43-3-e20190417-suppl7.pdf]

Supplementary Material to "*De novo* sequencing of *Bletilla striata* (Orchidaceae) transcriptome and identification of genes involved in polysaccharide biosynthesis"

Table S3 – Number of unigenes from *B. striata* assigned to KEGG reference pathways.

pathway	pathway_id	Gene_number
Ribosome	ko03010	529
Oxidative phosphorylation	ko00190	364
Protein processing in endoplasmic reticulum	ko04141	276
Spliceosome	ko03040	254
Glycolysis / Gluconeogenesis	ko00010	253
Purine metabolism	ko00230	222
RNA transport	ko03013	222
Starch and sucrose metabolism	ko00500	207
Plant hormone signal transduction	ko04075	179
Carbon fixation in photosynthetic organisms	ko00710	172
Ubiquitin mediated proteolysis	ko04120	163
Pyrimidine metabolism	ko00240	158
Pyruvate metabolism	ko00620	158
Amino sugar and nucleotide sugar metabolism	ko00520	153
Phagosome	ko04145	149
Arginine and proline metabolism	ko00330	147
Citrate cycle (TCA cycle)	ko00020	144
Plant-pathogen interaction	ko04626	144
Peroxisome	ko04146	142
Endocytosis	ko04144	138
Cysteine and methionine metabolism	ko00270	134
Ribosome biogenesis in eukaryotes	ko03008	124
mRNA surveillance pathway	ko03015	121
RNA degradation	ko03018	120
Alanine, aspartate and glutamate metabolism	ko00250	109
Pentose phosphate pathway	ko00030	105
Glutathione metabolism	ko00480	105
Photosynthesis	ko00195	99
Fructose and mannose metabolism	ko00051	97
Proteasome	ko03050	97
Aminoacyl-tRNA biosynthesis	ko00970	92
Valine, leucine and isoleucine degradation	ko00280	90
Fatty acid metabolism	ko00071	89
Phenylpropanoid biosynthesis	ko00940	86
Pentose and glucuronate interconversions	ko00040	81
Nucleotide excision repair	ko03420	81
Inositol phosphate metabolism	ko00562	78
Glyoxylate and dicarboxylate metabolism	ko00630	78
Phosphatidylinositol signaling system	ko04070	78
Glycerophospholipid metabolism	ko00564	75

pathway	pathway_id	Gene_number
Nitrogen metabolism	ko00910	75
Phenylalanine metabolism	ko00360	73
Protein export	ko03060	70
Ascorbate and aldarate metabolism	ko00053	68
Glycine, serine and threonine metabolism	ko00260	68
Propanoate metabolism	ko00640	68
Tryptophan metabolism	ko00380	67
DNA replication	ko03030	66
Glycerolipid metabolism	ko00561	65
Porphyrin and chlorophyll metabolism	ko00860	65
RNA polymerase	ko03020	65
Galactose metabolism	ko00052	64
Valine, leucine and isoleucine biosynthesis	ko00290	62
beta-Alanine metabolism	ko00410	62
N-Glycan biosynthesis	ko00510	58
Base excision repair	ko03410	57
Butanoate metabolism	ko00650	55
Biosynthesis of unsaturated fatty acids	ko01040	53
Photosynthesis - antenna proteins	ko00196	50
Terpenoid backbone biosynthesis	ko00900	49
Phenylalanine, tyrosine and tryptophan biosynthesis	ko00400	48
Homologous recombination	ko03440	48
Lysine degradation	ko00310	47
Basal transcription factors	ko03022	47
Tyrosine metabolism	ko00350	46
Mismatch repair	ko03430	46
Steroid biosynthesis	ko00100	37
Selenocompound metabolism	ko00450	37
Sphingolipid metabolism	ko00600	37
Pantothenate and CoA biosynthesis	ko00770	37
SNARE interactions in vesicular transport	ko04130	37
Histidine metabolism	ko00340	36
Flavonoid biosynthesis	ko00941	36
Natural killer cell mediated cytotoxicity	ko04650	36
Cyanoamino acid metabolism	ko00460	35
Carotenoid biosynthesis	ko00906	34
alpha-Linolenic acid metabolism	ko00592	32
Ubiquinone and other terpenoid-quinone biosynthesis	ko00130	30
Circadian rhythm - plant	ko04712	30
Limonene and pinene degradation	ko00903	29
One carbon pool by folate	ko00670	28
Sulfur metabolism	ko00920	28
Fatty acid biosynthesis	ko00061	26
Regulation of autophagy	ko04140	26
Ether lipid metabolism	ko00565	24
Tropane, piperidine and pyridine alkaloid biosynthesis	ko00960	24
Lysine biosynthesis	ko00300	23
ABC transporters	ko02010	22
Nicotinate and nicotinamide metabolism	ko00760	21
Folate biosynthesis	ko00790	21

pathway	pathway_id	Gene_number
Arachidonic acid metabolism	ko00590	20
Glycosylphosphatidylinositol(GPI)-anchor biosynthesis	ko00563	18
Isoquinoline alkaloid biosynthesis	ko00950	18
Circadian rhythm - mammal	ko04710	18
Synthesis and degradation of ketone bodies	ko00072	16
Other glycan degradation	ko00511	15
Stilbenoid, diarylheptanoid and gingerol biosynthesis	ko00945	15
Zeatin biosynthesis	ko00908	14
Flavone and flavonol biosynthesis	ko00944	14
Diterpenoid biosynthesis	ko00904	13
Taurine and hypotaurine metabolism	ko00430	12
Sulfur relay system	ko04122	12
Glycosaminoglycan degradation	ko00531	11
Vitamin B6 metabolism	ko00750	11
Non-homologous end-joining	ko03450	10
Linoleic acid metabolism	ko00591	9
Glycosphingolipid biosynthesis - globo series	ko00603	9
C5-Branched dibasic acid metabolism	ko00660	9
Thiamine metabolism	ko00730	9
Brassinosteroid biosynthesis	ko00905	9
Riboflavin metabolism	ko00740	8
Biotin metabolism	ko00780	8
Glycosphingolipid biosynthesis - ganglio series	ko00604	7
Fatty acid elongation in mitochondria	ko00062	4
Lipoic acid metabolism	ko00785	3
Caffeine metabolism	ko00232	2
Other types of O-glycan biosynthesis	ko00514	1
Indole alkaloid biosynthesis	ko00901	1
