# Supplementary material for: De novo sequencing of Bletilla striata (Orchidaceae) transcriptome and identification of genes involved in polysaccharide biosynthesis
Source: Genet Mol Biol. 2020 Jun 26;43(3):e20190417. doi: 10.1590/1678-4685-GMB-2019-0417 (PMC7315133; doi:10.1590/1678-4685-GMB-2019-0417)
Supplement: Supplementary file 3 [file 1415-4757-GMB-43-3-e20190417-suppl1.pdf]

Supplementary Material to "*De novo* sequencing of *Bletilla striata* (Orchidaceae) transcriptome and identification of genes involved in polysaccharide biosynthesis"

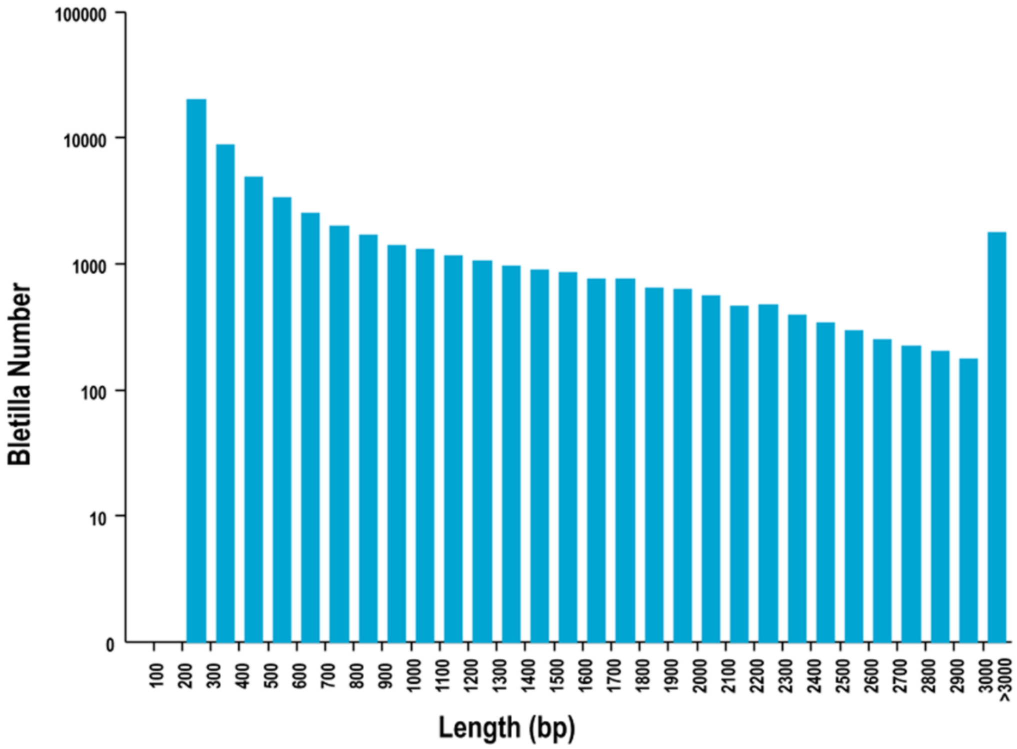

Figure S1 – Distribution of lengths for CDSs predicted from *B. striata* unigenes. Make BlastX comparisons of the unigenes of *B. striata* were made in NR, SwissProt, KEGG, and COG in turn. If the search in the first database gave a positive result, it was not queried against the next database. Otherwise, it will be automatically compared with the next protein database, until the end of the comparison was all databases. The CDSs predicted length is shown on the X-axis, and the Y-axis shows the number of CDSs.
