# Supplementary material for: De novo sequencing of Bletilla striata (Orchidaceae) transcriptome and identification of genes involved in polysaccharide biosynthesis
Source: Genet Mol Biol. 2020 Jun 26;43(3):e20190417. doi: 10.1590/1678-4685-GMB-2019-0417 (PMC7315133; doi:10.1590/1678-4685-GMB-2019-0417)
Supplement: Supplementary file 4 [file 1415-4757-GMB-43-3-e20190417-suppl8.pdf]

# Supplementary Material to "*De novo* sequencing of *Bletilla striata* (Orchidaceae) transcriptome and identification of genes involved in polysaccharide biosynthesis"

**Table S4 -** Identities of candidate unigenes related to BSP biosynthesis, analyzed at nucleotide level. Sequence similarities with each specified gene from *B. striata* (% Identity in last column) were calculated by the Basic Local Alignment Search Tool (BLAST) (http://blast.ncbi.nlm.nih.gov/).

A) *GMPP*

| Gene | Species | Accession number | Max score | Total score | Query cover | E-value | Identity |
|---|---|---|---|---|---|---|---|
| *DcGMPP1* | *Dendrobium catenatum* | XM_020826367.2 | 1524 | 1524 | 100% | 0 | 92.06% |
| *DcGMPP1* | *D. catenatum* | KP203853.1 | 1519 | 1519 | 100% | 0 | 91.97% |
| *PeGMPP* | *Phalaenopsis equestris* | XM_020734566.1 | 1203 | 1203 | 99% | 0 | 86.76% |
| *PdGMPP* | *Phoenix dactylifera* | XM_008803415.3 | 922 | 922 | 100% | 0 | 82.24% |
| *EgGMPP* | *Elaeis guineensis* | XM_029264871.1 | 881 | 881 | 100% | 0 | 81.38% |

B) *PMM*

| Gene | Species | Accession number | Max score | Total score | Query cover | E-value | Identity |
|---|---|---|---|---|---|---|---|
| *DcPMM1* | *D. catenatum* | XM_020824052.2 | 1170 | 1170 | 99% | 0 | 94.80% |
| *DoPMM* | *Dendrobium officinale* | KF195558.1 | 1158 | 1158 | 99% | 0 | 94.53% |
| *DhPMM* | *Dendrobium huoshanense* | KY912084.1 | 1155 | 1155 | 98% | 0 | 94.63% |
| *PePMM1* | *Phalaenopsis equestris* | XM_020725710.1 | 1096 | 1096 | 99% | 0 | 93.06% |
| *PePMM2* | *P. equestris* | XM_020725708.1 | 1096 | 1096 | 99% | 0 | 93.06% |
| *DcPMM2* | *D. catenatum* | XR_003700441.1 | 1068 | 1068 | 90% | 0 | 94.88% |
| *DcPMM3* | *D. catenatum* | XR_003700440.1 | 1068 | 1068 | 90% | 0 | 94.88% |
| *DcPMM4* | *D. catenatum* | XR_003700439.1 | 1068 | 1068 | 90% | 0 | 94.88% |
| *DcPMM5* | *D. catenatum* | XM_028694034.1 | 1068 | 1068 | 90% | 0 | 94.88% |
| *DcPMM6* | *D. catenatum* | XR_003700438.1 | 1068 | 1068 | 90% | 0 | 94.88% |
| *PePMM3* | *P. equestris* | XM_020725707.1 | 989 | 989 | 90% | 0 | 92.82% |

C) *manA*

| Gene | Species | Accession number | Max score | Total score | Query cover | E-value | Identity |
|---|---|---|---|---|---|---|---|
| *DcmanA1* | *Dendrobium catenatum* | XM_020847969.2 | 1567 | 1567 | 99% | 0 | 88.76% |
| *DcmanA2* | *D. catenatum* | XM_020847961.2 | 1567 | 1567 | 99% | 0 | 88.76% |
| *PemanA1* | *Phalaenopsis equestris* | XM_020723118.1 | 1472 | 1472 | 99% | 0 | 87.44% |
| *PemanA2* | *P. equestris* | XR_002293926.1 | 1472 | 1472 | 99% | 0 | 87.44% |
| *PemanA3* | *P. equestris* | XR_002293925.1 | 1472 | 1472 | 99% | 0 | 87.44% |

D) *HK*

| Gene | Species | Accession number | Max score | Total score | Query cover | E-value | Identity |
|---|---|---|---|---|---|---|---|
| *DcHK1* | *Dendrobium catenatum* | XM_020834402.2 | 776 | 776 | 100% | 0 | 92.74% |
| *PeHK1* | *Phalaenopsis equestris* | XM_020715628.1 | 726 | 726 | 100% | 0 | 91.08% |
| *PeHK2* | *P. equestris* | XM_020738746.1 | 577 | 577 | 78% | 2E-160 | 91.27% |
| *DcHK2* | *D. catenatum* | XR_002304111.2 | 182 | 353 | 44% | 2E-41 | 90.00% |

E) *scrK*

| Gene | Species | Accession number | Max score | Total score | Query cover | E-value | Identity |
|---|---|---|---|---|---|---|---|
| *DcscrK1* | *Dendrobium catenatum* | XM_020847040.2 | 2023 | 2023 | 98% | 0 | 91.45% |
| *DcscrK2* | *D. catenatum* | XM_028694480.1 | 1400 | 2039 | 98% | 0 | 90.62% |
| *DcscrK3* | *D. catenatum* | XM_028694479.1 | 1400 | 2039 | 98% | 0 | 90.62% |
| *DcscrK4* | *D. catenatum* | XM_028694481.1 | 736 | 1934 | 94% | 0 | 88.93% |
| *DcscrK5* | *D. catenatum* | XM_028694484.1 | 732 | 1731 | 83% | 0 | 88.89% |
| *DcscrK6* | *D. catenatum* | XM_028694482.1 | 671 | 1787 | 86% | 0 | 92.86% |

F) *GPI*

| Gene | Species | Accession number | Max score | Total score | Query cover | E-value | Identity |
|---|---|---|---|---|---|---|---|
| *DcGPI1* | *Dendrobium catenatum* | XM_020845619.2 | 2591 | 2591 | 99% | 0 | 94.13% |
| *PeGPI1* | *P. equestris* | XM_020734572.1 | 2399 | 2399 | 98% | 0 | 92.49% |
| *PeGPI2* | *P. equestris* | XM_020734575.1 | 2385 | 2385 | 98% | 0 | 92.32% |

| | | | | | | | |
|---|---|---|---|---|---|---|---|
| *PeGPI3* | *P. equestris* | XM_020734574.1 | 2385 | 2385 | 98% | 0 | 92.32% |
| *PeGPI4* | *P. equestris* | XM_020734573.1 | 2385 | 2385 | 98% | 0 | 92.32% |
| *PeGPI5* | *P. equestris* | XM_020734571.1 | 2385 | 2385 | 98% | 0 | 92.32% |
| *DcGPI2* | *D. catenatum* | XM_020845621.2 | 2172 | 2491 | 96% | 0 | 94.95% |

G) *pgm*

| Gene | Species | Accession number | Max score | Total score | Query cover | E-value | Identity |
|---|---|---|---|---|---|---|---|
| *Pepgm1* | *Phalaenopsis equestris* | XM_020730429.1 | 2383 | 2383 | 100% | 0 | 91.25% |
| *Dcpgm1* | *Dendrobium catenatum* | XM_020848783.2 | 2311 | 2311 | 100% | 0 | 90.51% |
| *Egpgm1* | *Elaeis guineensis* | XM_010927810.3 | 1489 | 1489 | 99% | 0 | 82.06% |
| *Egpgm2* | *E. guineensis* | XM_010927810.2 | 1489 | 1489 | 99% | 0 | 82.06% |
| *Aopgm1* | *Asparagus officinalis* | XM_020421571.1 | 1456 | 1456 | 98% | 0 | 82.02% |
| *Egpgm3* | *E. guineensis* | XM_010919195.3 | 1450 | 1450 | 98% | 0 | 81.83% |
| *Egpgm4* | *E. guineensis* | XM_010919195.2 | 1450 | 1450 | 98% | 0 | 81.83% |
| *Pdpgm1* | *Phoenix dactylifera* | XM_008777295.2 | 1378 | 1378 | 99% | 0 | 80.90% |
| *Pdpgm2* | *P. dactylifera* | XM_008777301.2 | 1341 | 1341 | 99% | 0 | 80.62% |
| *Nupgm1* | *Nelumbo nucifera* | XM_010272963.2 | 1304 | 1304 | 97% | 0 | 80.53% |
| *Nupgm2* | *N. nucifera* | XM_010267858.2 | 1301 | 1301 | 98% | 0 | 80.36% |
| *Akpgm1* | *Amorphophallus konjac* | JF727264.1 | 1299 | 1299 | 97% | 0 | 80.41% |

H) *UGP2*

| Gene | Species | Accession number | Max score | Total score | Query cover | E-value | Identity |
|---|---|---|---|---|---|---|---|
| *DcUGP2* | *Dendrobium catenatum* | XM_020818943.2 | 2244 | 2244 | 100% | 0 | 95.27% |
| *DoUGP21* | *Dendrobium officinale* | KF711982.1 | 2239 | 2239 | 100% | 0 | 95.20% |
| *DoUGP22* | *D. officinale* | JX294909.1 | 2222 | 2222 | 100% | 0 | 94.99% |
| *PeUGP2* | *Phalaenopsis equestris* | XM_020741275.1 | 2128 | 2128 | 100% | 0 | 93.79% |
| *AoUGP2* | *Asparagus officinalis* | XM_020417966.1 | 1182 | 1182 | 91% | 0 | 83.15% |
| *AcUGP2* | *Ananas comosus* | XM_020236732.1 | 1162 | 1162 | 90% | 0 | 83.09% |
| *NnUGP2* | *Nelumbo nucifera* | XM_010247886.2 | 1037 | 1037 | 98% | 0 | 80.13% |
