# Supplementary material for: De novo sequencing of Bletilla striata (Orchidaceae) transcriptome and identification of genes involved in polysaccharide biosynthesis
Source: Genet Mol Biol. 2020 Jun 26;43(3):e20190417. doi: 10.1590/1678-4685-GMB-2019-0417 (PMC7315133; doi:10.1590/1678-4685-GMB-2019-0417)
Supplement: Supplementary file 5 [file 1415-4757-GMB-43-3-e20190417-suppl2.pdf]

Supplementary Material to "*De novo* sequencing of *Bletilla striata* (Orchidaceae) transcriptome and identification of genes involved in polysaccharide biosynthesis"

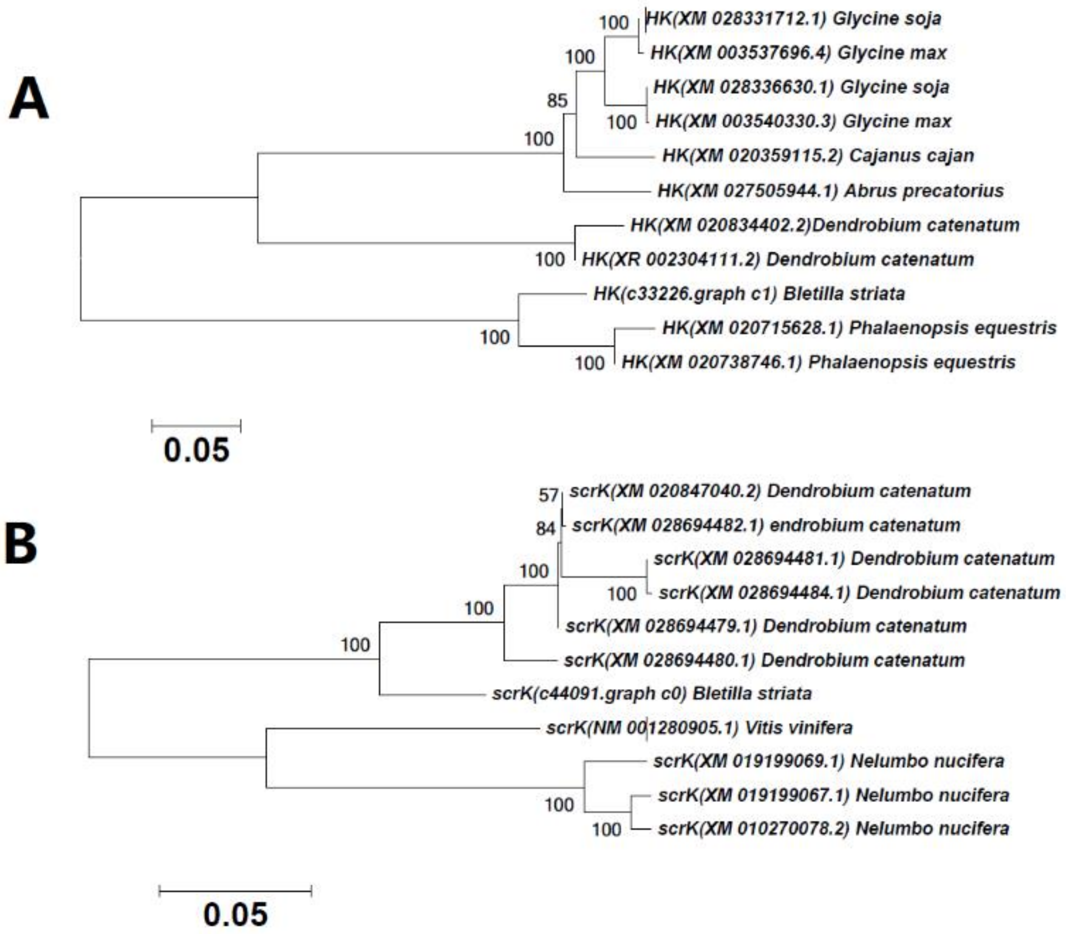

Figure S2 – Trees produced from Neighbor-joining phylogenetic analysis of homologs for *HK* (A) and *scrK* (B) in various plant species, as performed with MEGA 6 software.
