# Supplementary material for: De novo sequencing of Bletilla striata (Orchidaceae) transcriptome and identification of genes involved in polysaccharide biosynthesis
Source: Genet Mol Biol. 2020 Jun 26;43(3):e20190417. doi: 10.1590/1678-4685-GMB-2019-0417 (PMC7315133; doi:10.1590/1678-4685-GMB-2019-0417)
Supplement: Supplementary file 7 [file 1415-4757-GMB-43-3-e20190417-suppl4.pdf]

Supplementary Material to "*De novo* sequencing of *Bletilla striata* (Orchidaceae) transcriptome and identification of genes involved in polysaccharide biosynthesis"

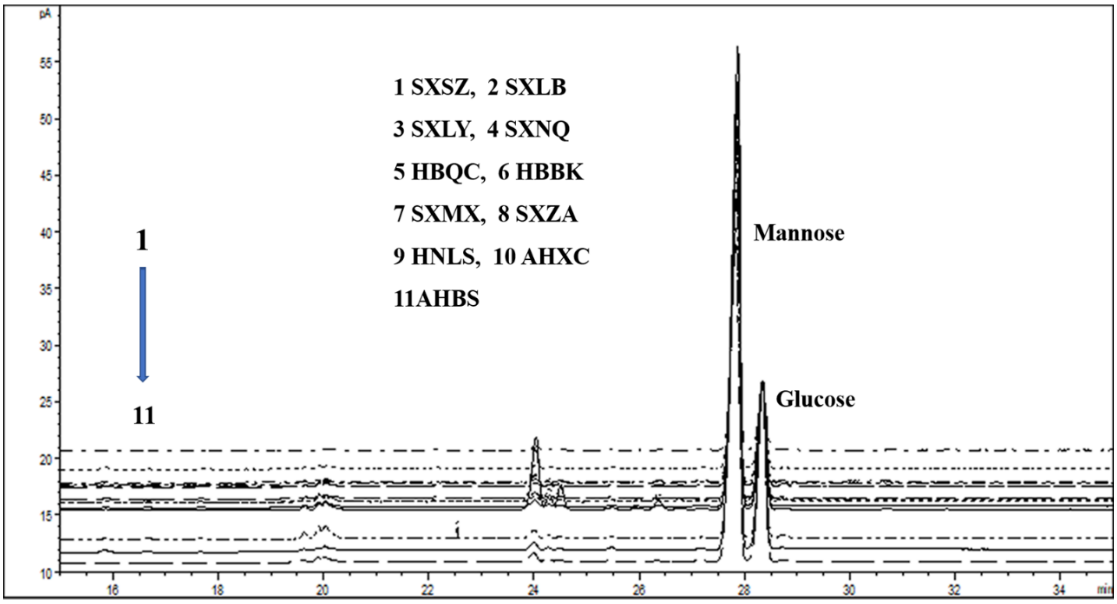

Figure S5 – GC-MS map of monosaccharide composition of BSP in different germplasm resources. Each dashed line represents a germplasm resource, and samples from SXSZ, SXLB, SXLY, SXNQ, HBQC, HBBK, SXMX, SXZA, HNLS, AHXC, AHBS are taken from top to bottom. The peak results of the samples from 11 regions show that they have a total of two peaks, representing mannose and glucose, respectively.
