# Supplementary material for: De novo sequencing of Bletilla striata (Orchidaceae) transcriptome and identification of genes involved in polysaccharide biosynthesis
Source: Genet Mol Biol. 2020 Jun 26;43(3):e20190417. doi: 10.1590/1678-4685-GMB-2019-0417 (PMC7315133; doi:10.1590/1678-4685-GMB-2019-0417)
Supplement: Supplementary file 8 [file 1415-4757-GMB-43-3-e20190417-suppl9.pdf]

# Supplementary Material to "*De novo* sequencing of *Bletilla striata* (Orchidaceae) transcriptome and identification of genes involved in polysaccharide biosynthesis"

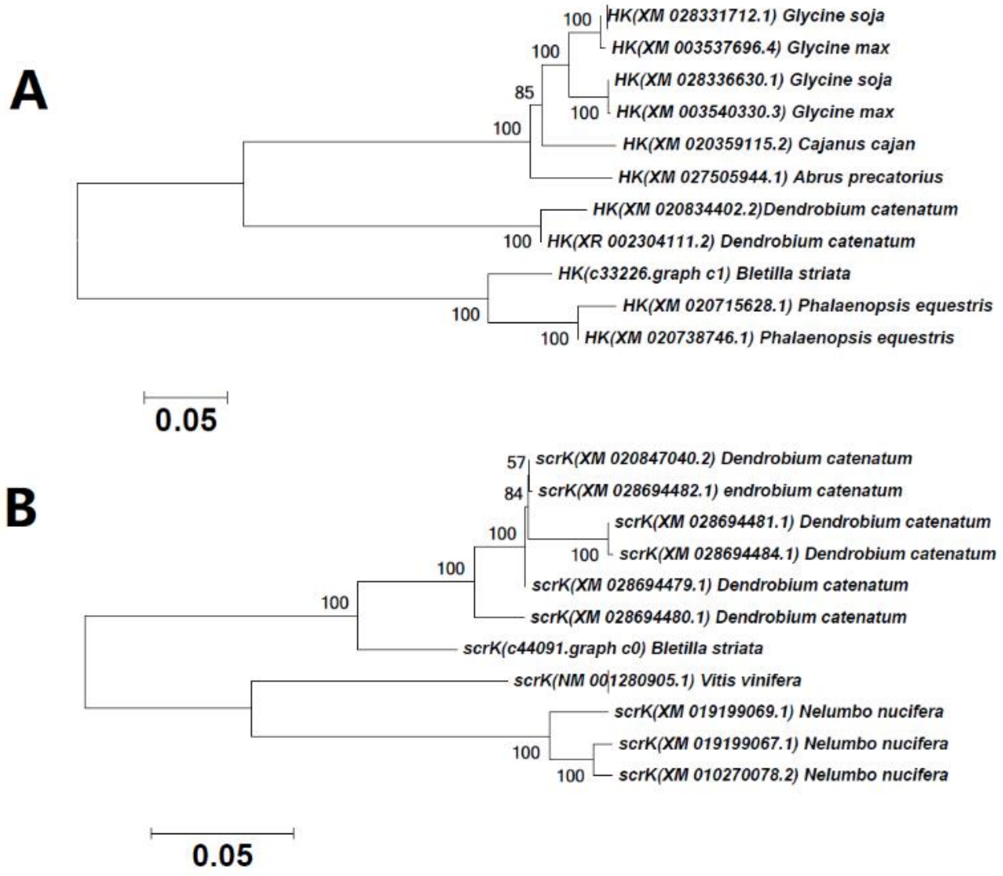


**Table S4** – Identities of candidate unigenes related to BSP biosynthesis, analyzed at nucleotide level. Sequence similarities with each specified gene from *B. striata* (% Identity in last column) were calculated by the Basic Local Alignment Search Tool (BLAST) (http://blast.ncbi.nlm.nih.gov/).
