# Supplementary material for: De novo sequencing of Bletilla striata (Orchidaceae) transcriptome and identification of genes involved in polysaccharide biosynthesis
Source: Genet Mol Biol. 2020 Jun 26;43(3):e20190417. doi: 10.1590/1678-4685-GMB-2019-0417 (PMC7315133; doi:10.1590/1678-4685-GMB-2019-0417)
Supplement: Supplementary file 9 [file 1415-4757-GMB-43-3-e20190417-suppl5.pdf]

Supplementary Material to "*De novo* sequencing of *Bletilla striata* (Orchidaceae) transcriptome and identification of genes involved in polysaccharide biosynthesis"

Table S1 – Origins of rhizome samples collected from different germplasms of *B. striata*.

Origin	Abbreviation	Longitude	Latitude	Elevation（m）
Shangzhou, Shaanxi province	SXSZ	109°48′18"	34° 2′6"	1,289
Liuba, Shaanxi province	SXLB	106°56′58″	33°43′27″	1,237
Lvyang, Shaanxi province	SXLY	106°25′32″	33°12′31″	1,342
Ningqiang, Shaanxi province	SXNQ	106°19′29″	33°3′39″	950
Qichun, Hubei province	HBQC	115°26'22.36"	30°13'53.72"	121
Baokang, Hubei province	HBBK	111°15'43.47"	31°52'53.88"	520
Mianxian, Shaanxi province	SXMX	106°40'28.03"	33° 9'40.65"	1,088
Zhenan, Shaanxi province	SXZA	109° 9′11"	33°25'33"	1,096
Lushi, Henan province	HNLS	111° 2'43.58"	34° 2'10.74"	882
Xuancheng, Anhui province	AHXC	118°45'25.52"	30°55'34.29"	33
Bashan, Anhui province	AHBS	117°38'19.16"	30°32'26.26"	77
